# Superconductivity in Ca-doped graphene laminates

**DOI:** 10.1038/srep23254

**Published:** 2016-03-16

**Authors:** J. Chapman, Y. Su, C. A. Howard, D. Kundys, A. N. Grigorenko, F. Guinea, A. K. Geim, I. V. Grigorieva, R. R. Nair

**Affiliations:** 1 School of Physics and Astronomy, University of Manchester, Manchester M13 9PL, UK; 2 Department of Physics and Astronomy, University College London, London, WC1E 6BT, UK; 3 Imdea Nanociencia, Faraday 9, 28015 Madrid, Spain

## Abstract

Despite graphene’s long list of exceptional electronic properties and many theoretical predictions regarding the possibility of superconductivity in graphene, its direct and unambiguous experimental observation has not been achieved. We searched for superconductivity in weakly interacting, metal decorated graphene crystals assembled into so-called graphene laminates, consisting of well separated and electronically decoupled graphene crystallites. We report robust superconductivity in all Ca-doped graphene laminates. They become superconducting at temperatures (*T*_c_) between ≈4 and ≈6 K, with *T*_c_’s strongly dependent on the confinement of the Ca layer and the induced charge carrier concentration in graphene. We find that Ca is the only dopant that induces superconductivity in graphene laminates above 1.8 K among several dopants used in our experiments, such as potassium, caesium and lithium. By revealing the tunability of the superconducting response through doping and confinement of the metal layer, our work shows that achieving superconductivity in free-standing, metal decorated monolayer graphene is conditional on an optimum confinement of the metal layer and sufficient doping, thereby bringing its experimental realization within grasp.

Graphene, a zero-gap semimetal, can be transformed into a metallic, semiconducting or insulating state by either physical or chemical modification^1^,^2^,^3^. Direct evidence for superconductivity is conspicuously missing among these states despite considerable experimental efforts as well as many theoretical proposals^4^,^5^,^6^. In contrast, nearly all allotropes of carbon including fullerenes, nanotubes, diamond and graphite were shown to exhibit superconductivity under heavy doping^7^,^8^,^9^,^10^. Interest in carbon-based superconductors has recently^11^,^12^,^13^ been revived by the discovery of superconductivity in CaC_6_, Ca-intercalated graphite compound (Ca-GIC) with *T*_c_ ≈ 11.5 K. Although some aspects of its superconductivity remain under debate^14^,^15^,^16^,^17^,^18^, main contributing factors have been identified^14^,^15^,^17^ as (i) doping via metal adatoms to reach sufficiently high electron concentrations in graphite, (ii) importance of an interlayer (IL) electronic band that arises from the intercalant superlattice formed between graphene layers, and (iii) the overall electron-phonon coupling that is related to coupling involving graphene phonons and intercalant vibrations. According to recent DFT calculations^4^, similar conditions are required to induce superconductivity in metal decorated graphene, i.e. adatoms are required not only to achieve sufficiently high electron concentrations, but also to create an electronic band arising from the adatom superlattice and ensure its overlap with the graphene π* band^4^. However, the effect of metal adatoms on free-standing graphene is predicted to be different from that in intercalated graphite. The difference is due to the quantum confinement of dopants’ wave functions in the latter: the absence of such confinement is expected to shift the IL band towards the Fermi level, thereby suppressing superconductivity in Ca-decorated graphene but enhancing it for Li doping^4^. Experimentally, previous attempts to induce superconductivity in monolayer graphene were limited to the proximity induced superconductivity^19^ and *in situ* ARPES measurements on metal decorated graphene^20^,^21^ which identified features attributed to dopant–related vibrational modes^20^ and found signatures of heavy doping as well as the appearance of an IL band in Ca-intercalated graphene bilayer (no IL band could be seen for Li intercalation). More recently, a superconducting gap was detected in Li-decorated monolayer graphene using ARPES measurements^22^ and the data used to suggest a *T*_*c*_ ~ 6 K. Due to the nature of these experiments, however, they could not provide direct evidence of the emergence of intrinsic superconductivity.

In this report, we have investigated the possibility of inducing superconductivity in graphene by decorating it with K, Cs, Li and Ca. To this end, we used so-called graphene laminates (GLs) that consist of graphene crystals arranged in a layered manner, similar to bulk graphite. However, unlike in graphite, crystallites within a GL are rotationally disordered and exhibit larger interlayer separations. This is known to result in effective decoupling of individual layers so that their electronic band structure corresponds to that of isolated graphene^23^. Accordingly, GLs offer a valuable alternative to individual graphene crystals in superconductivity studies because GLs can be produced in bulk and, therefore, measured using SQUID magnetometry, a method of choice for detecting superconductivity. In addition, bulk samples consisting of graphene and alkali monolayers are much less susceptible to environmental damage that arises due to extreme reactivity of alkali metals with oxygen, moisture, etc. We have employed different types of graphene laminates: those made directly from graphite (GLs), reduced graphene oxide laminates (RGOLs) and laminates containing both graphene and boron nitride (GBNLs). Samples were prepared using previously reported techniques^23^,^24^,^25^ (Methods). To insert metal atoms between graphene crystallites within the laminates we employed techniques similar to those used previously for graphite intercalation (Methods). The effect of metal insertion was immediately obvious from visual inspection. Similar to intercalated graphite^10^, GLs exhibited a pronounced colour change arising from changes in electronic structure upon doping (Fig. 1a–d). As discussed below, different colours of metal-intercalated GLs correspond to different plasmon energies due to different doping levels.

Figure 1e shows typical magnetisation vs temperature curves, *M*(*T*), for Ca-GL and Li-GL. Zero field cooling (ZFC) data for Ca-GL clearly shows a diamagnetic transition at ≈6.0 K (shielding of the external field, *H*, which is characteristic of superconducting materials). The onset transition temperature found from *H* = 0 *M*(*T*) curves is 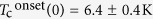, varying only slightly from sample to sample. The relatively broad superconducting transition is likely to be due to either different levels of doping for individual graphene crystallites or disorder in Ca monolayers (see below). In low *H* the samples clearly exhibit the Meissner effect (inset in Fig. 1e). Figure 1f shows the evolution of *M*(*T*) with increasing *H*: both the diamagnetic response and *T*_*c*_ decrease as expected. The *T*-dependent upper critical field, *H*_c2_(*T*) (see Supplementary Information) exhibits a positive curvature consistent with temperature-dependent critical fields for superconductors made of weakly coupled superconducting layers, such as alkali-metal intercalated MoS_2_ and Bi_2_Sr_2_CaCu_2_O_8_ 26. In contrast, neither K-, Cs- or Li-intercalated GLs showed any sign of a superconducting transition down to our lowest temperature of 1.8 K (red symbols in Fig. 1e and Supplementary information). Therefore, below we focus on Ca-decorated GLs only.

Further evidence for superconductivity in Ca-GLs was obtained from the temperature dependence of their electrical resistivity, *R*(*T*) (see Fig. 2a). The zero-field resistive transition is rather broad with an onset at *T*_c_ ≈ 12 K. The much higher *T*_*c*_ compared to that found in *M*(*T*) measurements indicates sample inhomogeneity, possibly due to the presence of some intercalated few-layer graphene (effectively ultrathin intercalated graphite; its *T*_c_ is expected to be similar to bulk graphite, 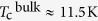
11,12). We emphasise that the fraction of few-layer graphene in our GLs is very small, and this higher-*T*_c_ phase could not be discerned in our *M*(*T*) measurements. The relatively small (factor of 2) drop in resistance is due to partial degradation of the sample: unlike all other techniques used in our study, transport measurements required relatively long air exposure of the samples before data could be taken. The resulting partial degradation of the samples was apparent from the colour change (see below) after the measurements; it was also apparent from magnetization measurements that showed an order of magnitude smaller diamagnetic moment compared to Fig. 1b.

To find out whether the observed superconducting response corresponds to a bulk layered system similar to intercalated graphite or, alternatively, is representative of superconductivity within individual graphene crystals, we have prepared mixed laminates where graphene crystallites are interspersed with BN flakes (GBNLs), and RGOLs where graphene flakes have larger separations compared to GLs, ≈3.6 Å versus ≈3.4 Å (Supplementary information). The mixed laminates were then intercalated with Ca using the same method as above. By adding extra BN layers in GBNLs, graphene crystallites were physically separated from each other. For example, in a 1:1 (weight) mixture of graphene and BN, statistically most of graphene crystals should have BN rather than graphene as its nearest neighbour. For higher concentrations of BN, graphene flakes are separated even further.

Ca intercalation of GBNLs and RGOLs was again evident from colour changes: In contrast to golden Ca-GL, Ca-RGOL is metallic brown whereas Ca-GBNLs’ colours varied from metallic brown to metallic green/blue with increasing BN content (Fig. 3a–d). We have found that Ca-RGOLs and Ca-GBNLs exhibit superconducting characteristics practically identical to those of Ca-GL, and the only pronounced difference is a reduction in *T*_c_ (Fig. 2b,c). Specifically, *T*_c_ for Ca-RGOL is reduced by ≈2 K and, for Ca-GBNLs, it decreases monotonically from ≈6.4 K to ≈4.4 K with increasing BN content up to 70%. Importantly, the addition of BN did not change either the width of the superconducting transition, or the superconducting fraction normalised to the graphene content (Fig. 2c). This strongly indicates that the superconductivity arises from independent Ca-decorated graphene crystals.

To understand the origin of different *T*_c_’s in Ca-GL, Ca-RGOL, and Ca-GBNLs and relate these to their electronic structures, we used X-ray analysis, Raman spectroscopy and optical reflectivity measurements to probe the laminates’ structure, phonons and plasmons, respectively. X-ray analysis revealed that the average separation of graphene layers in Ca-GLs and Ca-RGOLs is significantly larger than the interlayer spacing in the corresponding graphite intercalation compound (Ca-GIC): *d* ≈ 5.1 and 5.4 Å vs 4.5 Å (ref. 12), respectively, presumably due to weaker coupling between individual corrugated graphene crystallites within GLs. According to theory^4^, the IL band that forms as a result of metal deposition is sensitive to the separation between graphene layers, which changes dopants’ wavefunctions because of the quantum confinement. Furthermore, the increased *d* in Ca-GLs effectively reduces the overlap between the π*-band of graphene and the IL band, which should reduce both charge carrier concentration and electron-phonon interactions, thereby reducing *T*_c_ 4,27.

To estimate charge carrier concentrations, *n*, in Ca-GL, Ca-RGOL, Ca-GBNLs and compare them with that in Ca-GIC, we measured optical reflectivities of these compounds (Fig. 3e). The clear shift of the reflectivity minima to lower energies indicates a reduction in plasmon energy, *ω*_p_ (Supplementary information) or – equivalently – a reduction in the overall electron concentration, *n*. The plasmon energy is also related to the observed changes in visual colour of the compounds (cf. Figs 1 and 3). One can see that the reduction in *n* is accompanied by progressively lower *T*_c_ (Supplementary information).

Plasmon energies were determined by fitting the reflectivity curves with the expression for the reflection coefficient for metallic systems (Supplementary information). To extract 2D carrier concentrations from the measured *ω*_p_, we used a model where the metal-graphene layers are represented by electrostatically coupled two-dimensional units (Supplementary information). A similar model was successfully used in ref. 27 to explain the empirical correlation between the filling of the IL band and the occurrence of superconductivity in GICs. This yielded the following relation between *ω*_p_, electron concentrations in graphene and the IL band, *n*_c_ and *n*_IL_, and the layer separation *d*:


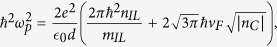


where *e* is the electron charge, *ϵ*_0_ the permittivity of free space, *v*_*F*_ the Fermi velocity in graphene and *m*_IL_ the mass of the metal ions. Unlike in a bulk metal where *ω*_p_ is determined only by the total carrier density, plasmon energies in layered systems also depend on the distribution of electrons between graphene and the IL band, and on *d*. For example, for Ca-GIC, Ca-GL, and Ca-RGOL, respectively, we obtain *n* ≈ 1.8 × 10^14^, 1.1 × 10^14^ and 9 × 10^13^ cm^−2^ (Supplementary information). According to the Bardeen-Cooper-Schrieffer (BCS) theory, such changes in *n* alone could in principle account for the observed differences in *T*_c_. For example, a ~10% reduction in *n* between Ca-GL and Ca-RGOL should reduce *T*_c_ by ~2 K (Supplementary information). This is in agreement with our observations (e.g., 6.4 K for Ca-GL and 4 K for Ca-RGOL). However, *ω*_p_ and *n* for Ca-GBNLs are lower than for Ca-RGOLs, in contrast to the opposite relation between their *T*_c_’s. Similar comparison between Ca-GIC and Ca-GL (~30% reduction in *n*, see supplementary information) suggests much larger suppression of *T*_c_ than observed. All this indicates that *n* is not the only factor at play. Furthermore, Li- and Ca- GLs had equal plasmon energies (Supplementary Table 1) and similar *n* but no superconductivity could be detected for Li-GL, similar to Li-GIC that is not superconducting^10^. The reason for so different superconducting properties of equally doped compounds has been suggested before^14^,^15^,^18^ as either occupied or unoccupied IL bands in Ca-GIC and Li-GIC, respectively. Our experiment highlights the fact that the same doping can result in different distributions of charge carriers between the graphene (Dirac) and IL bands. We believe that in the case of Ca-GL the IL band is occupied but for Li-GL it remains empty, similar to the case of Li-GIC.

Further information about relative contributions of Dirac and IL bands comes from Raman spectroscopy. For bulk Ca-GIC, Raman spectra are known^28^,^29^ to have two main features: an in-plane bond-stretching mode at ~1500 cm^−1^ and a weaker ~450 cm^−1^ mode due to out-of-plane vibrations. The latter originates from folding of the K-point graphene phonon to the Γ point in the larger unit cell defined by the √3 × √3 Ca superlattice. The 450 cm^−1^ mode has been shown to be sensitive to separation between graphene layers in different GICs^29^. As the layer separation increases, this mode blue-shifts, concomitant with the observed decrease in *T*_c_. We have found the out-of-plane mode for all the Ca-intercalated GLs (Fig. 3f) which confirms the presence of the √3 × √3 Ca superlattice (inset in Fig. 3f). The relatively broad peaks in Fig. 3f compared to Ca-GIC^28^ indicate notable disorder in the Ca superlattice, possibly at graphene edges. In contrast to Ca-GIC where the out-of-plane mode is at ≈440 cm^−1^, the corresponding Raman peaks for Ca-GL and Ca-RGOL are blue-shifted to ≈460 and 520 cm^−1^, respectively. A blue shift of this phonon mode compared to bulk GIC has been predicted^4^ for Ca-decorated monolayer graphene to occur due to a weaker confinement of the Ca layer. Our Raman data indicate the progressively weaker confinement from Ca-GIC to Ca-GL to Ca-RGOL, consistent with their increasingly larger interlayer distances: 4.5 Å to 5.1 Å to 5.4 Å, respectively. The position of the out-of-plane mode for Ca-GBNLs is similar to that of Ca-GLs (Fig. 3f) indicating comparable Ca confinement. Accordingly, the differences in *T*_c_ in these cases can be attributed to decreasing *n* with increasing BN content, as evident from the reflectivity measurements discussed above as well as from the corresponding blue shifts of the in-plane Raman mode (Supplementary information).

In conclusion, we have shown that graphene laminates doped with Ca exhibit robust superconductivity with a transition temperature governed by the electron transfer from the metal to graphene and by the Ca-layer confinement, as the latter is believed to dictate the overlap between the IL and graphene electronic bands. In contrast to theoretical predictions, no superconductivity could be detected above 1.8 K for the case of Li doping, possibly because the Li layer confinement in our laminates was still too strong. Curiously, as plasmon energies of Ca-doped graphene lie in the visible range, sample colours can be used as a simple guide to estimate *T*_c_.

## Methods

Pristine graphene laminates (GLs) were fabricated as reported earlier^23^,^24^,^30^. In brief, high purity HOPG crystals were exfoliated in N-Methyl-2-pyrrolidone (NMP) in an ultrasonic bath and the resulting dispersions centrifuged at 12,000 rpm to obtain a stable suspension. These were then filtered through porous alumina filters to obtain several μm (3–8 μm) thick free standing laminates of graphene. Reduced graphene oxide laminates were also prepared as reported previously^25^ (details in Supplementary information). Recent progress in reducing graphene oxide back to graphene^25^,^31^ allows synthesis of high quality RGO with few defects. In the present work GO was reduced using hydroiodic acid. GBNLs were prepared by the same method as GLs but with the filtration of composite solution of graphene-BN suspension in NMP (Supplementary information).

Metal intercalation was done in either high vacuum or an argon-filled glove box to avoid exposure of the highly reactive alkali-/alkali-earth metals and the intercalated samples to ambient moisture and oxygen. We have used both pure-metal vapour transport^10^,^11^ and alloy-intercalation techniques^12^ to insert K, Cs, Li and Ca into GLs (Supplementary information).

Magnetisation measurements were performed on 4 × 4 mm square samples using Quantum Design MPMS XL7 SQUID magnetometer (Supplementary information). In the zero field cooling (ZFC) mode, the samples were initially cooled to 1.8 K in zero applied field, then a desired external field *H* applied and the magnetisation *M* measured as a function of increasing temperature, *T* (typically 1.8–30 K). The field-cooling (FC) part of an *M*(*T*) curve was obtained on cooling the sample to 1.8 K in the same *H*.

For electrical transport measurements, we have fabricated GL devices in van-der-Pauw geometry, i.e. four contacts were made with silver paint in the corners of a 3 × 3 mm square sample of a graphene laminate. The devices were then intercalated with Ca using vapour transport technique, transferred to a container inside the glove box and quickly cooled down to liquid nitrogen temperature to avoid degradation. Later, the samples were transferred to a liquid helium cryostat, cooled down to 0.3 K and the resistance of the device continuously monitored while cooling. All transport measurements were performed using standard four probe DC measurement techniques using Keithley’s 2400 source-meter and 2182 A nanovoltmeter. Raman spectra were acquired using a Renishaw micro Raman spectrometer with a 514 nm excitation using a laser power <1 mW. Due to the extreme sensitivity of the samples to air, they were sealed inside quartz tubes in the inert atmosphere of a glove box to avoid degradation.

Optical reflectivity measurements were carried out using an Energetiq laser-driven light source (available wavelength range 190 nm–2.4 μm), where the light passed through a broadband fibre into a reflective collimator, a neutral density filter wheel and a 70/30 beam splitter before being focused with a 25× objective (NA – 0.65) onto the sample. To prevent degradation of the samples, they were covered with a thin film of paraffin oil and sealed inside a glass cell in the inert atmosphere of a glove box. To eliminate reflection from the glass plate encapsulating the sample, we used a refractive index matching gel. The sample was brought into focus with a three-dimensional stage manipulation system. The reflected light passed back through the lens and beam splitter, before being split again (92/8) to allow a digital image to be simultaneously captured by the camera (8%), with the remaining light (92%) then focused along another broadband fibre to the Ocean Optics spectrometer for analysis. The obtained digital images allowed us to confirm that the samples remained stable (did not degrade) during the measurements by monitoring their colour. The spectra were taken at 345–1040 nm wavelengths, using a silver mirror as a reference.

## Additional Information

**How to cite this article**: Chapman, J. *et al.* Superconductivity in Ca-doped graphene laminates. *Sci. Rep.* **6**, 23254; doi: 10.1038/srep23254 (2016).

## Supplementary Material

Supplementary Information

## Figures and Tables

**Figure 1:**
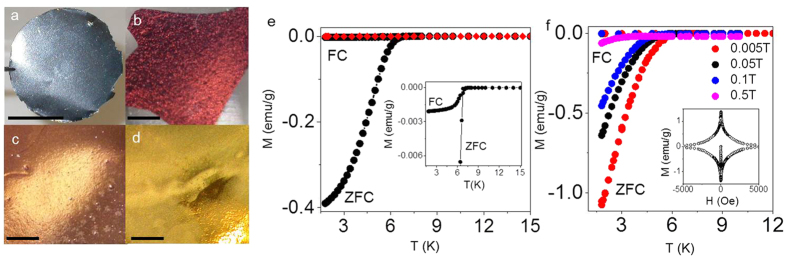
Characterisation of intercalated graphene laminates. (**a**–**d**) Optical photographs of pristine (scale bar 1 cm) and K-, Cs- and Ca- intercalated GLs (scale bar 1 mm), respectively. Li-GL (not shown) has a similar colour to Ca-GL. (**e**) Temperature dependence of ZFC and FC mass magnetisation, *M*, for Li-GL (red symbols) and Ca-GL (black) at *H* = 4 Oe applied parallel to the laminates’ surface. The estimated systematic error in determining *M* is ~10% due to inaccuracy of measuring the sample mass that was typically several mg (this is difficult because of the extreme sensitivity of intercalated GLs to moisture and oxygen). The inset shows a zoom-up of the *M*(*T*) curve for Ca-GL. (**f**) Main panel: ZFC and FC *M*(*T*) at different *H* for Ca-GL. The inset shows the magnetisation dependence as a function of *H* ‖ ab at 1.8 K, which is characteristic of type-II superconductors with significant trapping of magnetic flux (pinning). The particularly strong flux trapping (very small FC Meissner response) is likely to be due to the fact that our laminates are made up from thousands of individual graphene crystallites, with inevitable voids between them.

**Figure 2:**
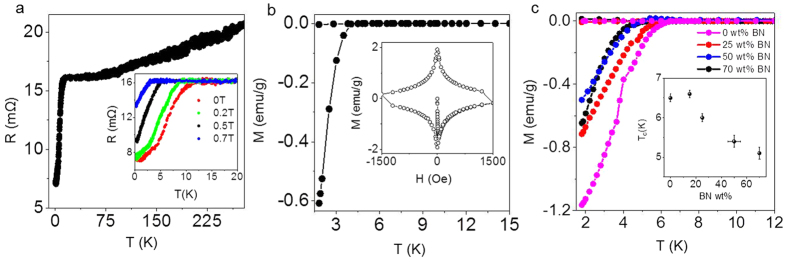
Superconductivity in Ca-GL, Ca-RGOL and Ca-GBNLs. (**a**) Temperature dependence of the electrical resistivity of a 3 μm thick 3 × 3 mm sample of Ca-GL showing a superconducting transition at ≈12 K. The inset shows the evolution of *R*(*T*) with increasing external magnetic field, *H*. The sample did not reach zero-resistance state, probably as a result of partial degradation because of brief exposure to air during transfer into a cryostat (Methods). (**b**) Temperature dependence of ZFC and FC magnetisation for Ca-RGOL at 4 Oe applied parallel to the graphene plane. The inset shows an example of the corresponding *M*(*H*); *T* = 1.8 K. (**c**) Magnetisation of Ca-GBNLs with different BN contents (*M* is normalised to the graphene content, i.e. only the mass of graphene is included for each GBNL). The inset shows the dependence of *T*_c_ on BN concentration (weight %).

**Figure 3:**
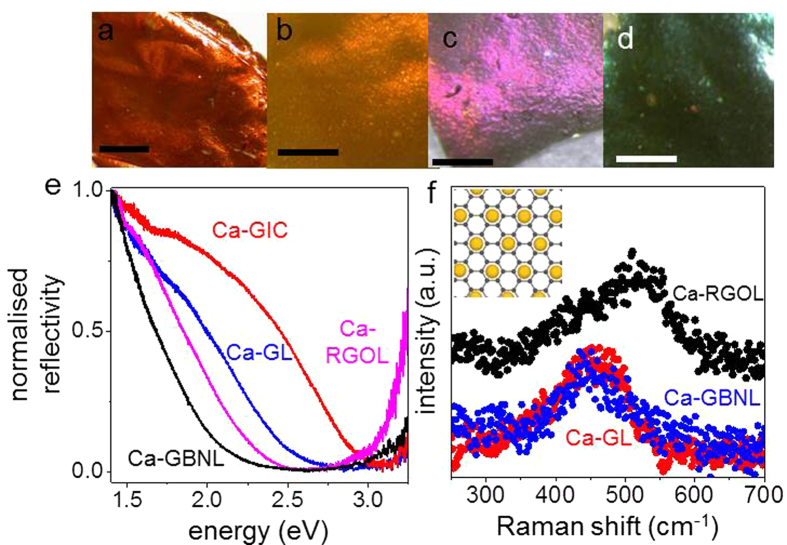
Optical characterisation of different Ca-doped laminates. (**a**–**d**) Photographs of Ca-RGOL and Ca-GBNLs with 25, 50 and 70 wt% BN content, respectively. Scale bars, 1 mm. (**e**) Reflectivity spectra for Ca-intercalated graphite and graphene laminates. (**f**) Out-of-plane Raman mode for different laminates. Ca-GBNL (blue) had 50 wt% of BN. The inset shows a sketch of the Ca superlattice on graphene; large yellow circles are Ca atoms and small black dots are carbon atoms.
