# Supplementary material for: Superconductivity in Ca-doped graphene laminates
Source: Sci Rep. 2016 Mar 16;6:23254. doi: 10.1038/srep23254 (PMC4793231; doi:10.1038/srep23254)
Supplement: Supplementary Information [file srep23254-s1.pdf]

Supplementary Information

Superconductivity in Ca-doped graphene laminates

J. Chapman[1], Y.Su[1], C. A. Howard[2], D. Kundys[1], A. N. Grigorenko[1], F. Guinea[1], A. K. Geim[1], I.V. Grigorieva[1*], R. R. Nair[1*]

[1]School of Physics and Astronomy, University of Manchester, Manchester M13 9PL, UK

[2]Department of Physics and Astronomy, University College London, London, WC1E 6BT, UK

*irina@manchester.ac.uk, rahul@manchester.ac.uk

Preparation of reduced graphene oxide laminates (RGOLs)

To produce graphite oxide we used high-purity crystals of highly-oriented pyrolytic graphite (HOPG). The crystals were broken into small pieces and oxidised using a modified Hummers' method[1], with all oxidation reactions carried out below 10 °C to minimise formation of defects during the reactions. This method has been shown to produce – after chemical reduction - high quality graphene with lower amounts of defects than conventional Hummer's method[2], as demonstrated by high carrier mobilities in corresponding devices (>1000 $cm^2V^{-1}s^{-1}$)[1]. Graphite oxide flakes were exfoliated in water in an ultrasonic bath and then centrifuged at 10000 rpm to separate monolayer graphene oxide (GO) flakes. The high degree of hydrophilicity of GO ensures very efficient exfoliation with nearly 100% yield of graphene oxide monolayers[3]. By adjusting sonication parameters we were able to controllably vary the typical size of individual GO crystallites between ~ 0.2 and 20 μm, but did not notice any effect of the crystallite size on the superconducting properties (after Ca intercalation). GO laminates (GOLs) were prepared from GO dispersions by filtration through alumina membranes with 20 nm pore size. As an additional measure to ensure the absence of metallic impurities in the samples prior to intercalation[4], all GOLs were immersed in concentrated HNO_3 for 24h before and after the chemical reduction.

To convert graphene oxide laminates into graphene laminates (RGOLs) we used chemical reduction[5,6] with hydroiodic (HI) acid. This method has been shown to produce higher quality RGO films and laminates, with fewer defects, than other reduction methods[7]. Reduction was carried out by immersing GO laminates in HI acid for 30 minutes, followed by repeated rinsing with ethanol to remove residual HI.

Intercalation of graphene-based laminates and bulk graphite with K, Cs, Li and Ca

To decorate graphene crystallites in the laminates with K, Cs and Li, we used a technique similar to the well-established vapour transport method[8]. To this end, a sample of GL and the chosen metal were placed inside a tantalum foil envelope and transferred to a stainless steel or glass tube sealed with a valve, all of this done in the high-purity argon atmosphere inside a glove box. The tube was then evacuated to ≈ 10^{-6} mbar and the whole assembly heated in a furnace to an appropriate temperature (200°C for K and Cs and 350°C for Li) in order to vaporise the metal. The high vacuum in the reaction container was maintained by continuous pumping to prevent oxidation of the reactants or the products. After approximately 40 hours of heating / exposure to the metal vapour, the products were recovered in a dry argon atmosphere. In the case of Li intercalation of GLs, we found it necessary to use a lower temperature compared to intercalation of bulk graphite reported in literature[9] (350 ^{o}C vs 400 ^{o}C): at 400 ^{o}C graphene partially reacted with Li to form lithium carbide that could be detected as an additional peak in the corresponding X-ray spectra. No carbides were formed in Li-GLs at 350 ^{o}C. The reference bulk graphite samples (Li-GIC) were intercalated at 400 ^{o}C and did not show any lithium carbide signals in the X-ray data, in agreement with literature. For K and Cs intercalation, we used the same temperature (200 ^{o}C) for both GLs and the reference bulk graphite.

For Ca intercalation, we used both vapour transport[10,11] and alloy techniques[12]. In the former case, a GL sample was placed alongside an ingot of calcium metal inside a quartz tube evacuated to <10^{-7} mbar and degassed at 350 ^{o}C for 24 hours in a set up similar to that in ref. [11]. The temperature was then increased to 470 ^{o}C in order to vaporise Ca metal. After exposing the GL sample to Ca vapour for one to two weeks, the Ca-GL sample was recovered from the container and stored in a dry argon atmosphere inside a glove box. For comparison, we have also prepared Ca-decorated mechanically exfoliated monolayer graphene and reference samples of Ca intercalated bulk graphite (Ca-GIC), using the same vapour transport method. To this end, a ~40 x 20 µm graphene monolayer was exfoliated onto an oxidised Si substrate by micromechanical cleavage and identified by optical contrast and Raman spectroscopy[13,14]. It was then exposed to Ca vapour with different exposure times (to vary the Ca coverage) and characterised using Raman spectroscopy (see below). Successful Ca coating of the graphene monolayer was also noticeable from an increase in optical contrast, similar to the colour changes observed for Ca-GLs.

An alternative intercalation method, the so-called alloy technique used previously for intercalation of Ca into bulk graphite[12], was employed to insert Ca into some of the GLs and all RGOLs and graphene-BN mixed laminates (GBNLs). To this end, GLs, RGOLs and GBNLs were exposed to molten calcium-lithium (≈ 20 at.% Ca) alloy at ~350 ^{o}C under dry argon atmosphere of a glove box for 12-18 hours. In addition to intercalation of Ca between the graphene crystallites in the laminate, this left a thin layer of metal on the sample surface, which was subsequently removed by gentle

scratching with a ceramic scalpel. The superconducting properties (T_c, evolution of magnetisation with field and temperature) as well as Raman signatures of Ca-GLs prepared using the two different techniques (vapour transport and alloy intercalation) were identical, in agreement with earlier experiments on Ca-GIC[10,12].

Magnetisation measurements

Magnetic response of metal-intercalated samples was measured using a commercial SQUID magnetometer Quantum Design MPMS XL7. To prevent degradation of the samples during transfer to the cryostat and subsequent measurements, all samples were immersed in paraffin oil and sealed inside polycarbonate capsules in the dry argon atmosphere of a glove box, then quickly transferred to the cryostat and immediately cooled down to below ~30K. The superconducting response of Ca-intercalated laminates is discussed in detail in the main text. In contrast to Ca intercalation, no superconductivity could be detected in Li-, K- and Cs intercalated GLs down to 1.8K (the lowest available temperature). Supplementary Fig. S1a shows ZFC and FC *M*(*T*) for Li-GLs. The weak paramagnetic response obvious in Fig. S1a is not discernible on the scale of Fig. 1e in the main text where it is also shown as red symbols. Similar purely paramagnetic behaviour was observed for Cs-GLs (not shown). The high level of electron doping in Li- and Cs-GLs was evident from the emergence of Pauli paramagnetism – linear, temperature-independent *M(H)* (Supplementary Fig. S1b) but it did not result in the emergence of superconductivity. For K-GLs, the magnetic behaviour was more complex (inset in Fig. S1a) showing hysteresis between ZFC and FC *M*(*T*), possibly related to coupling between weakly magnetic K clusters, as suggested in ref. [15].

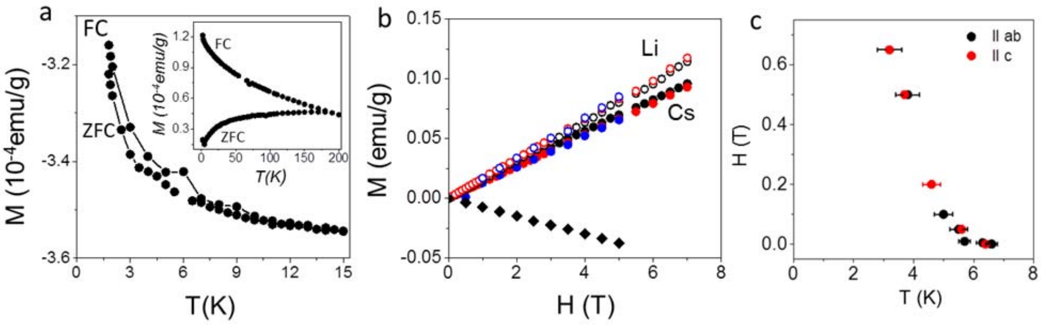

Supplementary Figure S1. *(a) Main panel: ZFC and FC temperature-dependent magnetisation of Li-GL; H = 50 Oe. Inset: same for K-GL at H= 10 Oe. (b) Magnetic-field dependent magnetisation of Cs-GL and Li-GL showing a linear, temperature-independent response: black symbols: T=1.8 K; red symbols: T=10 K and blue symbols: T=100 K. As a reference, black diamonds show the diamagnetic response of GL before intercalation. (c)* Phase diagram for Ca-GL obtained from the *M*(*T*) plots for different *H* applied parallel (black dots) and perpendicular (red) to the graphene plane.

The upper critical field, $H_{c2}(T)$, of Ca-GLs was estimated from ZFC measurements of H-dependent critical temperature, T_c^{onset}; the corresponding phase diagram is shown in Fig. S1c. The total absence of anisotropy is surprising however, we note that the shown $H_{c2}(T)$ values should be treated as approximate, rather than exact values, as ZFC *M(T)* curves correspond to diamagnetic screening. The latter is known to create significant gradients of the magnetic field from the outer surfaces to the centre of the samples, leading to an uncertainty in H to which different Ca-decorated graphene crystallites within a laminate sample were exposed. Nevertheless, the measurements clearly indicate a positive curvature in $H_{c2}(T)$ as expected for weakly coupled superconducting layers[16].

GBN mixed laminates

Crystals of h-BN purchased from Manchester Nanomaterials Ltd[17] were exfoliated in N-Methyl-2-pyrrolidone (NMP) using ultra-sonication as reported previously[18]. The dispersions were centrifuged at 12,000 rpm three times in order to remove multilayer flakes and to obtain a stable suspension of BN. After that, composite graphene-BN suspensions were prepared by mixing graphene and BN suspensions in a desired proportion, followed by further ultra-sonication. A similar technique was used recently to prepare artificial van der Waals solids with electrical, mechanical, and optical properties distinctly different from those of the 'parent' layers[19]. Graphene-BN laminate samples (GBNLs) were then prepared in the same way as GLs, i.e. by filtration through an alumina membrane. The finished laminates were characterised using X-ray diffraction and Raman spectroscopy (see below). X-ray diffraction patterns for GBNLs with different BN content were similar to the pristine GLs, yielding the same layer spacing, $d \approx 3.4$Å. Such similarity is to be expected due to the nearly identical crystal lattices of h-BN and graphite. Intercalation of GBNLs with Ca was done using the alloy method as described above.

X-ray diffraction

To determine the interlayer separation in different graphene-based laminates we used X-ray diffraction. Similar measurements were used previously to measure the interlayer separation in Ca-GIC, which was found to be ≈ 4.5 Å[10,12]. Due to the sensitivity of Ca-GLs and Ca-RGOLs samples to air, they were sealed inside an airtight specimen holder transparent to X-rays (purchased from Bruker). Even with this protection, the environmental stability of the Ca-laminates was poorer than for intercalated bulk graphite, e.g., after multiple repeated scans the new peaks due to Ca insertion gradually disappeared, concomitant with a re-appearance of the peaks characteristic for pristine GLs. Therefore, all measurements were done as quickly as possible.

Supplementary Figure S2a,b highlights the shift in X-ray diffraction peaks corresponding to the interlayer separation in GL and RGOL before and after Ca intercalation. Before Ca insertion the interlayer separations in GLs and RGOLs were,

respectively, $d \approx$ 3.3-3.5 Å and 3.6-3.8 Å (Fig. S2a). The larger interlayer separation in RGOLs compared to GLs could be due to the GO reduction mechanism: During chemical reduction, oxygen-containing functional groups are removed as water or gas molecules and the release of these molecules from the interior of the laminate can induce corrugation and larger interlayer spacing compared to GLs[6]. The same diffraction peaks after Ca insertion is shown in Fig. S2b: the new peak positions correspond to $d \approx$ 5.1 Å for Ca-GL and ≈ 5.4 Å for Ca-RGOL, i.e. the interlayer spacing in Ca-RGOL is still ≈0.3 Å larger that in Ca-GL, a similar difference as for the corresponding pristine samples. We note that the above diffraction peaks become markedly narrower after Ca intercalation (cf. panels (a) and (b) in Fig. S2). The reason for this is not clearly understood and difficult to investigate due to gradual sample degradation but may be an indication that the formation of Ca superlattice between corrugated and misaligned graphene flakes makes the layer structure more ordered in the direction perpendicular to the layers.

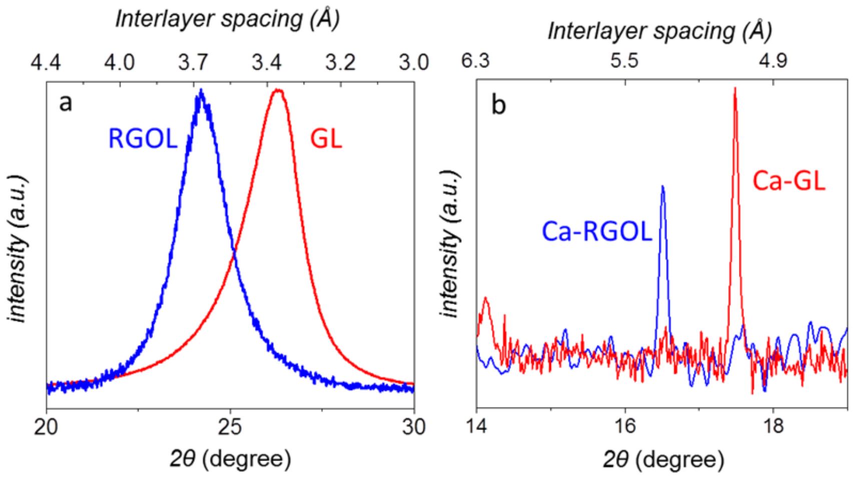

Supplementary Figure S2. *X-ray diffraction peaks corresponding to the layer separation (a) in pristine GL and RGOL and (b) in Ca-GL and Ca-RGOL (background from the sample holder subtracted).*

Raman spectroscopy

Raman spectroscopy has been shown to permit qualitative understanding of the phonons and the degree of doping of graphene layers in intercalated graphite[11,20]. In particular, the level of doping of mono- and few-layer graphene exposed to K metal vapour was found to be continuously tunable due to increasing coverage with K atoms, until saturation is reached after a number of repeated exposures[11]. Such tunability was in contrast to bulk GICs that form distinct stoichiometric compounds[8].

In our work, we used Raman spectroscopy to probe the out-of-plane graphene phonons, as well as the level of doping, and to investigate the differences between Ca-GIC, Ca-GLs and the Ca-decorated monolayer graphene (MLG) exfoliated onto an oxidised silicon substrate (see above). Supplementary Figure S3a shows the evolution of Raman spectra for the MLG as Ca coverage is increased through

repeated exposures to Ca vapour. For pristine graphene, the spectrum shows the expected single-component 2D band at ~ 2690 cm^{-1} and the G-peak at ~ 1580 cm^{-1}. After intermediate Ca exposures, the 2D peak first decreases in intensity and then disappears altogether, due to raising of the Fermi level and the removal of the resonance conditions. This is accompanied by significant broadening and a red shift of the G-peak that takes on a Breit-Wigner-Fano (BWF) line shape. Similar softening and broadening of the Raman modes have been observed previously on monolayer graphene decorated with K and were interpreted as evidence of high doping and increased electron-phonon interactions[11]. Some new features also appear between 1200 cm^{-1} and 1400 cm^{-1}, similar to those found in K decorated monolayer graphene[11].

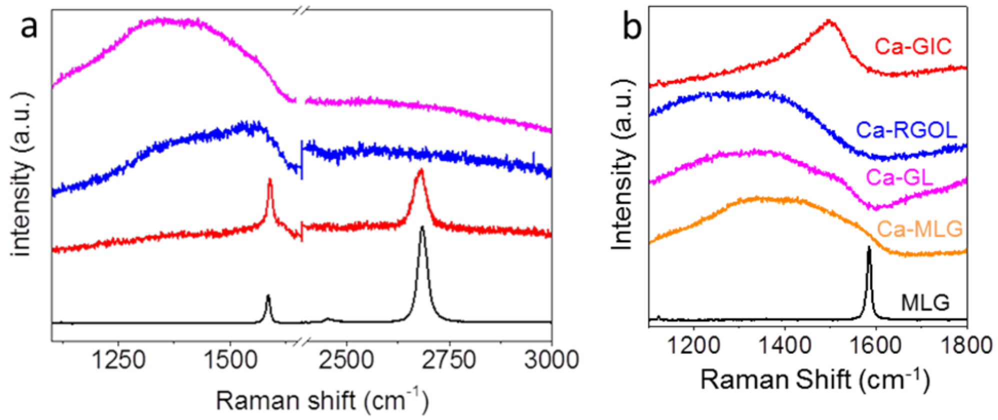

Supplementary Figure S3. *(a) Raman spectra of a monolayer graphene (MLG) exfoliated onto an oxidised Si substrate and repeatedly exposed to Ca vapour. Black curve: pristine state; red and blue curves: intermediate exposures; magenta curve: saturated Ca coverage. (b) Comparison of the Raman spectra for different Ca-decorated samples.*

The shape and position of the Raman G-peak for Ca-GL, Ca-RGOL and Ca-MLG are compared in Supplementary Fig. S3b. For comparison, we also show the Raman spectra of the pristine graphene (before exposure to Ca), and of the bulk Ca-GIC. In agreement with earlier measurements[21,22], the Raman spectrum for our Ca-GIC has a single BWF-shaped peak at ~ 1500 cm^{-1}, due to the Raman active E_g mode corresponding to the in-plane bond stretching vibrations in graphene layers. The larger width and the red shift of this peak with respect to pristine graphite (where G peak is found at ~1580 cm^{-1}) are believed to be due to an increase in electron-phonon interaction at large doping[11,22].

As clear from Fig. S3b, the Raman spectra of Ca-intercalated GL and Ca-RGOL are significantly more similar to the spectrum of the Ca-saturated MLG than to bulk Ca-GIC, indicating similar electronic properties of these three systems. These spectra serve as another indication that Ca-decorated graphene crystallites in Ca-GLs and Ca-RGOLs are effectively independent from each other and exhibit the same characteristics as similarly doped monolayer graphene.

Gradual doping and colour change.

To investigate whether the doping level of graphene-based laminates can be tuned continuously, as in the case of the monolayer, or the exposure to Ca vapour/molten Li-Ca alloy results in the formation of a stoichiometric CaC_6 as in bulk graphite[10,12], we varied the time of exposure of GLs to Ca, Li, K and Cs metals. (We note that only the fully saturated first stage CaC_6 was found to exist in Ca-GIC; unlike Li-and K-intercalation[8], to the best of our knowledge a lower stage CaC_6 compound has not been reported). Supplementary Figure S4 compares optical photographs of intercalated laminates after the exposure corresponding to saturation (such that no further colour changes occurred with further increase of exposure time) and after carrying out the intercalation process for approximately half the time (top and bottom rows, respectively). It is clear that the colours of GLs exposed to a metal for a shorter time are different from those intercalated to saturation, indicating lower carrier concentrations (see below), presumably due to lower coverage of graphene crystallites with metal atoms. Accordingly, it should be possible, in principle, to continuously tune the level of doping and the associated electronic properties of graphene laminates, similar to an isolated MLG[11]. In practice however, at intermediate exposures it was difficult to achieve uniform colours (that is, uniform metal coverage and doping) over an entire GL sample; the colours shown in the bottom row of Fig. S4 were only found in some parts of a sample while other parts were either still dark grey (not intercalated) or of a yet another colour. Achieving an intermediate coverage was particularly difficult for Ca intercalation, as is clear from comparison of the corresponding images in Fig. S4.

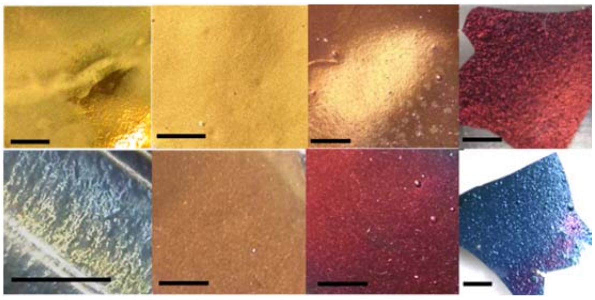

Supplementary Figure S4. *Optical photographs of fully- and partially intercalated GLs (top and bottom rows, respectively). The metals used for intercalation, from left to right: Ca, Li, Cs and K. For example, the top left image is Ca-GL intercalated to saturation and bottom left image is Ca-GL after twice shorter intercalation time. All scale bars correspond to 1mm.*

Therefore we used an alternative approach and varied the carrier concentration by using mixed graphene-BN laminates (GBNLs), as described above, and exposing them to Ca metal until saturation was achieved. The colours of GBNLs with different BN content are shown in Fig. 3 of the main text. Corresponding Raman spectra (Supplementary Fig. S5) clearly indicate a decrease of the carrier density, n, in Ca-coated graphene crystallites within GBNLs as the proportion of BN in a laminate is

increased (see also the reflectivity spectrum in Fig. 3e and the discussion below). Raman spectra taken on many different parts of each of these samples were identical, verifying sample homogeneity. Comparison of the three spectra in Supplementary Fig. S5 shows that the addition of 25% BN did not have a significant effect on *n*, with the same broad peak observed at ~1400 cm^{-1} as for Ca-GL and Ca-RGOL (Supplementary Fig. S3). In contrast, the spectra for 50% and 70% BN show signatures of much less doped graphene (G peak at ≈1550-1600 cm^{-1}) and of hBN (1365 cm^{-1} peak[23]). The lower level of Ca doping (presumably due to lower coverage with Ca atoms) is also evident from optical reflectivity measurements (main text and see below).

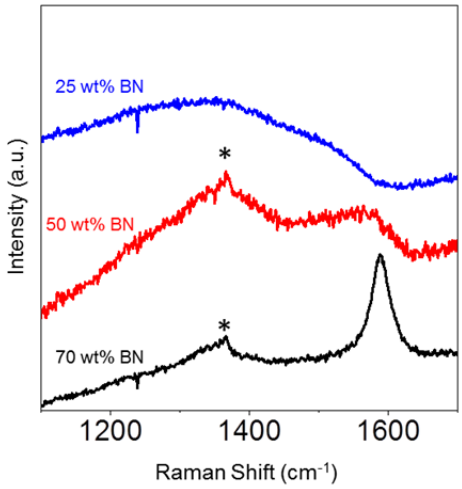

Supplementary Figure S5. *Raman spectra of Ca-GBNLs with 25, 50 and 70 wt% BN content (* indicates the peak corresponding to hBN).*

<u>Optical reflectivity.</u>

In addition to the reflectivity data presented in the main text, spectra were also taken for Li-, K- and Cs-intercalated GLs – see Supplementary Fig. S6a. To extract information about the electronic properties of the system (e.g., plasma frequency), we fit the experimental spectra with the well-known expression for the reflection coefficient, $R = \left|\frac{n-1}{n+1}\right|^2$, where the refractive index $n^2 \approx k\left(1 - \frac{\omega_p^2}{\omega(\omega - i/\tau)}\right)$ is derived from Maxwell's equations[24]. Here, *k* is the dielectric permittivity of the environment, ω_p the plasma frequency, ω the frequency of the incident light and τ the electron collision time. The relatively shallow slopes of *R*(*E*) curves in Figs. 3 and S6 indicate inhomogeneity of the electron distribution in the samples (for example due to slightly different coverage of individual graphene crystallites with metal atoms or different coverage at the edges of crystallites). To account for this, the fitting procedure allowed for a variation of ω_p within ±0.3-0.5 eV range – see caption to Fig. S6 for exact fitting parameters. As an example, Supplementary Figure S6b shows the experimental reflectivity spectrum for Ca-GL and the corresponding fitting curve; the extracted plasma frequency in this particular case was ω_p = 2.6 eV. Similar analysis

was carried out for all other samples; the results are given in Supplementary Table 1. Notably, while Ca-GLs are superconducting and Li-GLs are not, both have the same plasmon energy, indicating similar overall electron concentrations. GLs intercalated with Cs and K also have plasmon energies similar to each other but significantly lower than for Ca- and Li-intercalated laminates. Compared to samples of intercalated bulk graphite that we prepared in parallel with GLs, all metal-doped GLs have significantly lower plasmon energies, again indicating lower overall electron concentrations in all laminate samples compared to intercalated graphite, presumably due to larger separations between graphene and metal atoms in the laminates compared to GICs.

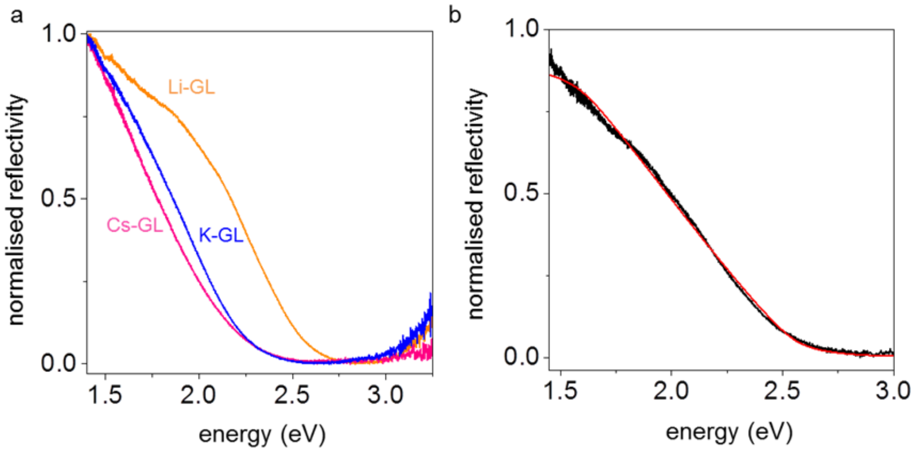

Supplementary Figure S6*. (a) Normalised reflectivity spectra of Li-, Cs- and K-intercalated GLs. (b) A normalised reflectivity spectrum of Ca-GL and the corresponding data fitting for the reflection coefficient, R(E).* The fitting parameters in this case were: $\Delta\omega_p$ =0.5 eV; τ =0.1*; k* =2.

Sample	plasmon energy, *E* (eV)
Ca-GL	2.6
Ca-RGOL	2.4
Ca-GBNL (50 wt%)	2.1
Li-GL	2.6
Cs-GL	2.3
K-GL	2.3
Ca-GIC	3.1
Li-GIC	3.0
K-GIC	2.5
Cs-GIC	2.5

Supplementary Table 1. *Plasmon energies of different intercalated GLs and corresponding bulk GICs estimated from the reflectivity spectra.*

Calculations of charge carrier distribution in metal-intercalated graphene laminates

For a layered multicomponent system, such as metal-intercalated GLs, the knowledge of ω_p alone is not sufficient to extract quantitative information about the density of carriers and their distribution between graphene and the metal layers. To achieve this, we used a simple model where the metal-graphene layers are represented by a set of two two-dimensional units coupled electrostatically[25] and calculated the carrier densities in graphene and the metal layer (Inter layer, IL), such that they correspond to the experimentally found ω_p. The electronic structure within each unit is given by three degenerate Dirac bands and one parabolic band located between the graphene and the metal layer[25]. A related situation, plasmons in a system consisting of a graphene layer and a two dimensional electron gas, has been considered in ref. [26]. Besides the electronic bands, other parameters included in the model are the width of the graphene layer, d_C, the width of the region occupied by the interlayer state, d_{IL}, the dielectric constant of the background, ϵ_0, and the total number of carriers, n_{total} - see Supplementary Fig. S7. The constraint of charge neutrality implies that the charge in the metal, n_{total}, satisfies $n_{total} = -n_C - n_{IL}$, where n_C and n_{IL} are the carrier densities in graphene and in the IL band, respectively.

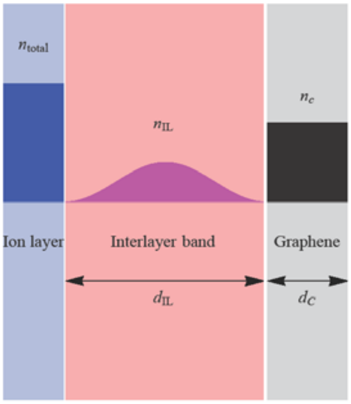

Supplementary Figure S7. *Schematic representation of metal decorated graphene layers in GLs.*

The electronic bands (Supplementary Fig. S8) are

$$\varepsilon_D(\vec{k}) = E_D + v_F|\vec{k}|$$
$$\varepsilon_{IL}(\vec{k}) = E_{IL} + \frac{\hbar^2|\vec{k}|^2}{2m_{IL}}.$$

We take the energy at the Dirac point as $E_D = 0$. The minimum of the interlayer band is E_{IL} and the Fermi energy is $E_F > E_{IL}, E_D$, so that the parabolic band is partially occupied, and the Dirac bands are *n* doped.

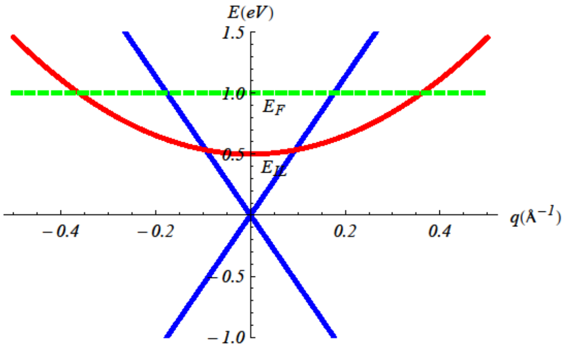

Supplementary Figure S8. *Sketch of the electronic bands for metal decorated layers of graphene. Blue: three-fold degenerate graphene bands; red: interlayer band; green: Fermi level.*

The properties of each metal-graphene compound in our study are determined by the total number of carriers, n_{total}. From n_{total} the number of carriers in the graphene layer, n_C, and the IL band, n_{IL}, are derived by minimizing the total energy. Once the carrier distribution is determined, we calculate the plasmon frequency, ω_p. The value of n_{total} is then adjusted so that (i) the plasmon frequency agrees with our experimental reflectivity data and (ii) the value of n_{IL} is zero in non-superconducting compound (Li-GL) and it is a monotonically increasing function of the superconducting critical temperature, T_c, in Ca-GLs, Ca-RGOL, Ca-GIC, as observed in our experiments. Due to the complexity of modelling a multilayer system containing both graphene and BN, we did not perform calculations for Ca-GBNLs.

As found experimentally, Ca-GL and Li-GL have the same plasmon energy, $\hbar\omega_p \cong 2.6$ eV. We estimate d_{IL} and d_C from the experimentally determined interlayer separations, $d_{IL} + d_C$, in proportion to the known atomic radii of carbon and the dopant metal. For example, for Ca-GL $d_{IL} + d_C \approx$ 5.1Å, $d_{IL} \approx 3.7$ Å and $d_C \approx 1.4$ Å; for Li-GL $d_{IL} + d_C \approx$ 3.7Å, $d_{IL} \approx 2.5$ Å and $d_C \approx 1.2$ Å . We also consider Ca-GIC and Ca-RGOL where the interlayer distances are $d_{IL} + d_C \approx 4.5$ Å and 5.4Å, respectively, and the corresponding plasmon energies, $\hbar\omega_p$= 3.1 eV and 2.4 eV.

The distribution of carriers between the interlayer and graphene bands is determined by the electrostatic interactions, the quantum capacitance of each band and the position of E_{IL} (Fig. S8). We assume that the charge in graphene, n_C, is distributed uniformly within a layer of thickness d_C,

$$\rho_C(x) = \begin{cases} 0 & x \leq -d_C \\ \dfrac{n_C}{d_C} & -d_C \leq x \leq 0 \\ 0 & 0 \leq x \end{cases}$$

The distribution of the charge in the IL band is

$$\rho_{IL}(x) = \begin{cases} 0 & x \leq 0 \\ \frac{n_{IL}}{2d_{IL}} \sin^2\left(\frac{\pi x}{d_C}\right) & 0 \leq x \leq d_{IL} \\ 0 & d_{IL} \leq x \end{cases}$$

The charge of the metal, n_{total}, is within a thin layer at $x = d_{IL}$.The electrostatic interactions per unit area between the metal ions and the carriers in graphene and the interlayer band are:

$$E_{el}^{ions-C} = e^2 n_C n_{total} \frac{d_C + 2d_{IL}}{16\pi}$$

$$E_{el}^{ions-IL} = e^2 n_{IL} n_{total} \frac{d_{IL}}{32\pi}$$

The electrostatic interactions between carriers are

$$E_{el}^{C-C} = e^2 n_C^2 \frac{d_C}{24\pi}$$

$$E_{el}^{IL-IL} = e^2 n_{IL}^2 \frac{d_{IL}(-3 + 2\pi^2)}{96\pi^2}$$

$$E_{el}^{C-IL} = e^2 n_C n_{IL} \frac{d_C + d_{IL}}{32\pi}$$

Finally, the quantum contribution of the carriers is:

$$E_q^C = \frac{2\hbar v_F}{\pi}\left(\frac{\pi n_C}{3}\right)^{3/2}$$

$$E_q^{IL} = E_{IL} n_{IL} + \frac{\hbar^2 \pi n_{IL}^2}{2m_{IL}}$$

The values of n_C and n_{IL} minimize the total energy

$$E_{tot} = E_{el}^{ions-C} + E_{el}^{ions-IL} + E_{el}^{IL-IL} + E_{el}^{C-C} + E_{el}^{C-IL} + E_q^C + E_q^{IL}$$

with a constraint $n_{total} = -n_C - n_{IL}$. For $E_{IL} > 0$ and small values of n_{total} all carriers reside in the graphene layer. As n_{total} increases, carriers move into the IL band.

The total carrier concentrations were optimised as shown by labels in Supplementary Fig. S9, e.g., $\cong 1.8 \times 10^{14}$ cm^{-2} for, Ca-GIC, 1.1×10^{14} cm^{-2} for Ca-GL and so on. Fig. S9 shows the total energy for different modelled structures as a function of n_C, at fixed n_{total}. We assume that the position of the interlayer band does not vary significantly among the different compounds. We choose $E_D = 0$, and $E_{IL} = 0.5$ eV, so that, at low total carrier density the carriers are in the graphene layer, and move into the interlayer band as the concentration increases.

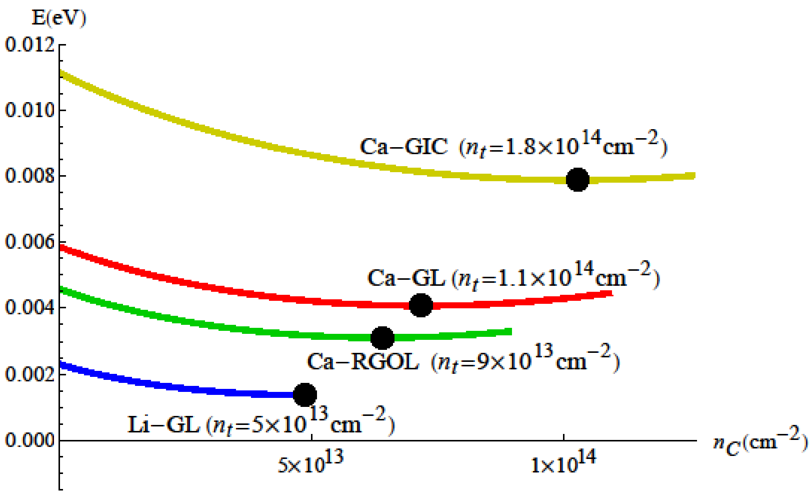

Supplementary Figure S9. *Total energy of the modelled structures as a function of the carrier density in graphene layers. Black dots indicate the minimum total energy and the labels give corresponding values of n_{total} with ~20% accuracy. The Fermi velocity of the Dirac bands is that of graphene, and the effective mass of the parabolic band is $m_{IL} = m_e$. The remaining parameters are given in the text.*

Minimization of E_{tot} allowed us to find the distribution of carriers between graphene and the IL bands. The obtained contributions of n_{total} to the graphene bands are ≈ 1.0×10^{14}, 7.2×10^{13} and 6.4×10^{13} cm^{-2} for the modelled Ca-GIC, Ca-GL and Ca-RGOL, respectively, i.e. bulk Ca-GIC has approximately 30% higher electron concentration in graphene layers compared to Ca decorated graphene crystallites in GLs.

The plasmon energy for one graphene-IL band unit can be calculated from the polarization, $\chi(\vec{q},\omega)$. At small wave vectors $\vec{q}$ it is

$$\chi(\vec{q},\omega) = 3 \times \chi_C(\vec{q},\omega) + \chi_{IL}(\vec{q},\omega) \approx \frac{3v_F k_F^C |\vec{q}|^2}{\pi\hbar\omega^2} + \frac{2(k_F^{IL})^2|\vec{q}|^2}{2\pi m_{IL}\omega^2}$$

The plasmon frequency, ω_p, is given by the solution of the equation

$$1 = v_{\vec{q}}\, \chi\left(\vec{q},\omega_p^2\right)$$

where $v_{\vec{q}} = (2\pi e^2)/(\epsilon_0|\vec{q}|)$ is the Coulomb potential, and

$$n_{IL} = \frac{(k_F^{IL})^2}{2\pi}$$

$$n_C = \frac{3(k_F^C)^2}{\pi}$$

In terms of the carrier densities n_C and n_{IL}, the plasmon frequency is

$$\hbar^2\omega_p^2(\vec{q}) = \frac{e^2|\vec{q}|}{\epsilon_0}\left(\frac{2\pi\hbar^2 n_{IL}}{m_{IL}} + 2\sqrt{3\pi}\hbar v_F\sqrt{|n_C|}\right)$$

This calculation can then be extended to a periodic stack of the two-dimensional graphene-IL units. In this case the plasmon frequency obeys

$$1 = v_{\vec{q}}\, \chi\left(\vec{q}, \omega_p^2\right) \frac{\sinh(|\vec{q}|d)}{\cosh(|\vec{q}|d) - \cos(k_z d)}$$

where d is the spacing of the units and k_z is the wavevector along the direction normal to the units. For $|\vec{q}|d, k_z d \rightarrow 0$ the plasmon frequency is

$$\hbar^2\omega_p^2 = \frac{2e^2}{\epsilon_0 d}\left(\frac{2\pi\hbar^2 n_{IL}}{m_{IL}} + 2\sqrt{3\pi}\hbar v_F \sqrt{|n_C|}\right)$$

This expression shows that the plasmon frequency depends not only on the total carrier density (as would be the case for a 3D metal), but also on how the carriers are distributed between the graphene layer and the IL band. Once the carrier distribution is determined by minimizing the energy (Fig. S9), the plasmon frequencies can be calculated using the above expression. Supplementary Fig. S10 shows the dependence of plasmon energy, $\hbar\omega_p$, on the carrier concentration in the IL band, n_{IL}.

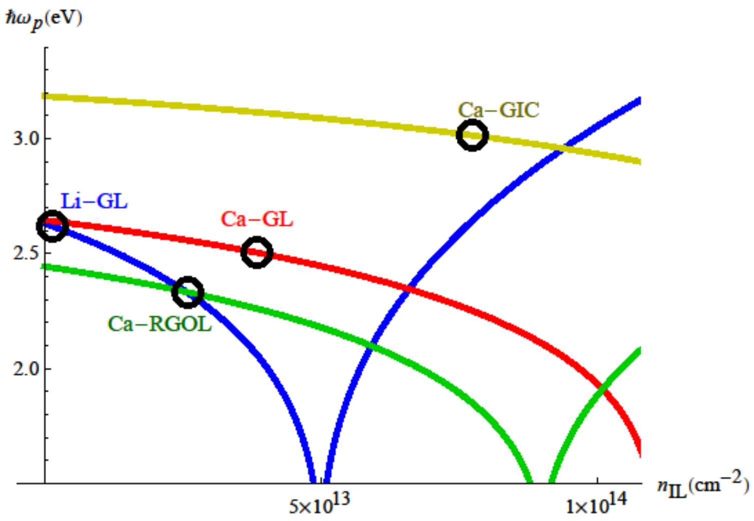

***Supplementary Figure S10**. Plasmon energy of an infinite stack of layers with one parabolic band and three Dirac bands as a function of the carrier density in the interlayer band, n_{IL}. The round symbols indicate the value of n_{IL}corresponding to the experimentally determined plasmon energy.*

Analysis of Fig. S10 clearly shows that it is possible for different metal-intercalated compounds to have the same plasmon energy (as is the case for Li-GL and Ca-GL) but different distributions of charge carriers between the IL and graphene bands, and as a result different superconducting properties. Furthermore, for superconducting metal-intercalated graphene compounds, their T_c depends on the level of doping of the graphene layers, which, in its turn, is determined by the total density of charge carriers in the system. That is, compounds with lowest total carrier densities (in our case, RGOLs and GBNLs with 70% BN) are also expected to have lowest carrier

concentrations in graphene layers and, accordingly, lowest T_c's, in agreement with the observations.

Finally, we can use the estimated differences in carrier concentrations in different superconducting samples (Ca-GIC, Ca-GLs and Ca-RGOLs) to estimate the changes in the critical temperature, T_c, that would be expected if this were the only factor affecting T_c. As was shown in ref. [27], variations in the density of states, $\delta N(0)$ lead to variations in T_c according to

$$\frac{\delta T_c}{T_{c0}} = \frac{1}{N(0)V}\frac{\delta N(0)}{N(0)},$$

where

$$N(0)V = -1/\ln\left(\frac{T_{c0}}{1.14\theta_D}\right).$$

Here T_{c0} is the initial T_c, θ_D is the Debye temperature and V the electron-phonon coupling strength. As the density of states of the interlayer band does not depend on the filling, we can assume that $\delta N(0)$ is proportional to variations of the carrier concentration in doped graphene layers, i.e.

$$\frac{\delta N(0)}{N(0)} \approx \frac{\Delta n_c}{n_c}$$

or

$$\Delta T_c(\Delta n) \approx T_{c0}\frac{\Delta n_c}{n_c}\ln\left(\frac{T_{c0}}{1.14\theta_D}\right)$$

Using θ_D= 175K[28] and $\Delta n_c/n_c \approx 10\%$ calculated as shown above from the measured plasmon energies for Ca-GL and Ca-RGOL, we expect $T_c^{\text{Ca-RGOL}}$ to be 2.2K lower than $T_c^{\text{Ca-GL}}$, in excellent agreement with the observed difference of 2.4K. However, a similar comparison of Ca-intercalated bulk graphite ($n_C \approx 1.0 \times 10^{14}$ cm^{-2}) and Ca-GL ($n_C \approx 7.2 \times 10^{13}$ cm^{-2}) gives a large overestimate of the expected decrease in T_c, indicating that other factors must be taken into account as explained in the main text.

Supplementary references

1 Eigler, S. *et al.* Wet Chemical synthesis of graphene. Adv. Mater.**25,** 3583-3587 (2013).

2 Hummers, W. S. & Offeman, R. E. Preparation of graphitic oxide. *J. Am. Chem. Soc.* **80,** 1339-1339 (1958).

3 Eda, G. & Chhowalla, M. Chemically derived graphene oxide: towards large-area thin-film electronics and optoelectronics. *Adv. Mater.* **22,** 2392-2415 (2010).

4 Sepioni, M., Nair, R. R., Tsai, I. L., Geim, A. K. & Grigorieva, I. V. Revealing common artifacts due to ferromagnetic inclusions in highly oriented pyrolytic graphite. *Europhys. Lett.* **97,** 47001 (2012).

5 Pei, S. & Cheng, H.-M. The reduction of graphene oxide. *Carbon* **50,** 3210-3228 (2012).

6 Chua, C. K. & Pumera, M. Chemical reduction of graphene oxide: a synthetic chemistry viewpoint. *Chem. Soc. Rev.* **43,** 291-312 (2014).

7 Su, Y. *et al.* Impermeable barrier films and protective coatings based on reduced graphene oxide. *Nature commun.* **5,** 4843 (2014).

8 Dresselhaus, M. S. & Dresselhaus, G. Intercalation compounds of graphite. *Adv. Phys.* **51,** 1-186 (2002).

9 Guerard, D. & Herold, A. Intercalation of lithium into graphite and other carbons. *Carbon* **13,** 337-345 (1975)

10 Weller, T. E., Ellerby, M., Saxena, S. S., Smith, R. P. & Skipper, N. T. Superconductivity in the intercalated graphite compounds C_6Yb and C_6Ca. *Nature Phys.* **1,** 39-41 (2005).

11 Howard, C. A., Dean, M. P. M. & Withers, F. Phonons in potassium-doped graphene: The effects of electron-phonon interactions, dimensionality, and adatom ordering. *Phys. Rev. B* **84,** 241404 (2011).

12 Emery, N. *et al.* Superconductivity of Bulk CaC_6. *Phys. Rev. Lett.* **95,** 087003 (2005).

13 Blake, P. *et al.* Making graphene visible. *Appl. Phys. Lett.* **91,** 063124 (2007).

14 Ferrari, A. C. *et al.* Raman spectrum of graphene and graphene layers. *Phys. Rev. Lett.* **97,** 187401 (2006).

15 Takai, K. *et al.* Magnetic potassium clusters in a nanographite host system. *Phys. Rev. Lett.* **98,** 017203 (2007).

16 Klemm, R. A. *Layered Supercondictors*, Chapter 2. (Oxford University Press, oxford 2012).

17 www.manchesternanomaterials.com.

18 Nicolosi, V., Chhowalla, M., Kanatzidis, M. G., Strano, M. S. & Coleman, J. N. Liquid exfoliation of layered materials. *Science* **340,** 1226419 (2013).

19 Gao, G. *et al.* Artificially stacked atomic layers: Toward new van der waals solids. *Nano Lett.* **12,** 3518-3525 (2012).

20 Das, A. *et al.* Monitoring dopants by Raman scattering in an electrochemically top-gated graphene transistor. *Nature Nanotechnol.* **3,** 210-215 (2008).

21 Hlinka, J. *et al.* Lattice dynamics of CaC_6 by Raman spectroscopy. *Phys. Rev. B* **76,** 144512 (2007).

22 Dean, M. P. M., Howard, C. A., Saxena, S. S. & Ellerby, M. Nonadiabatic phonons within the doped graphene layers of XC_6 compounds. *Phys. Rev. B* **81,** 045405 (2010).

23 Gorbachev, R. V. *et al.* Hunting for monolayer boron nitride: Optical and raman signatures. *Small* **7,** 465-468 (2011).

24 Chazalviel, J.-N. *Coulomb Screening by Mobile Charges: Applications to Materials Science, Chemistry, and Biology*. (Birkhäuser Boston 1999)

25 Boeri, L., Bachelet, G., Giantomassi, M. & Andersen, O. Electron-phonon interaction in graphite intercalation compounds. *Phys. Rev. B* **76,** 064510 (2007).

26 Principi, A., Carrega, M., Asgari, R., Pellegrini, V. & Polini, M. Plasmons and Coulomb drag in Dirac-Schrodinger hybrid electron systems. *Phys. Rev. B* **86,** 085421 (2012).

27 Staley, N. E. *et al.* Electric field effect on superconductivity in atomically thin flakes of $NbSe_2$. *Phys. Rev. B* **80,** 184505 (2009).

28 Jobiliong, E. *et al.* Anisotropic superconductivity in bulk CaC_6. *Phys. Rev. B* **76,** 052511 (2007).
